# Appraising epidemiology data and antimicrobial resistance of urinary tract infections in critically ill adult patients: a 7-year retrospective study in a referral Brazilian hospital

**DOI:** 10.1590/1516-3180.2021.0933.R1.24022023

**Published:** 2023-05-12

**Authors:** Vitelhe Ferreira de Almeida, Maria Clara Bisaio Quiliici, Sebastiana Silva Sabino, Daiane Silva Resende, Iara Rossi, Paola Amaral de Campos, Rosineide Marques Ribas, Paulo Pinto Gontijo-Filho

**Affiliations:** I MSc. Biologist and Doctoral Student, Institute of Biomedical Sciences, Laboratory of Molecular Microbiology, Universidade Federal de Uberlândia (UFU), Uberlândia (MG), Brazil.; II MSc. Nurse, Institute of Biomedical Sciences, Laboratory of Molecular Microbiology, Universidade Federal de Uberlândia (UFU), Uberlândia (MG), Brazil.; III MSc. Nurse and Doctoral Student Institute of Biomedical Sciences, Laboratory of Molecular Microbiology, Universidade Federal de Uberlândia (UFU), Uberlândia (MG), Brazil.; IV PhD. Biologist, Institute of Biomedical Sciences, Laboratory of Molecular Microbiology, Universidade Federal de Uberlândia (UFU), Uberlândia (MG), Brazil.; V PhD. Biologist, Institute of Biomedical Sciences, Laboratory of Molecular Microbiology, Universidade Federal de Uberlândia (UFU), Uberlândia (MG), Brazil.; VI PhD. Biologist, Institute of Biomedical Sciences, Laboratory of Molecular Microbiology, Universidade Federal de Uberlândia (UFU), Uberlândia (MG), Brazil.; VII PhD. Biologist and Full Professor, Department of Microbiology, Universidade Federal de Uberlândia (UFU), Uberlândia (MG), Brazil.; VIII PhD. Retired Professor, Department of Microbiology, Universidade Federal de Uberlândia (UFU), Uberlândia (MG), Brazil.

**Keywords:** Chain of infection, Bacterial zoonoses, Urinary tract infections, Epidemiology, Catheter-associated urinary tract infection, Etiology, Multi-resistant

## Abstract

**BACKGROUND:**

Urinary tract infections (UTI) are highly preventable and have significant clinical and financial impact on the patient and the health care system.

**OBJECTIVE:**

To investigate UTIs in critically ill adult patients and the relationship of antimicrobial consumption and multidrug-resistant isolate.

**DESIGN AND SETTING:**

A cohort study performed in a Brazilian tertiary-care university hospital in the city of Uberlandia (MG), located at the Federal University of Uberlandia, southeast region of the country.

**METHODS:**

We analyzed a cohort of 363 patients with first episode of UTIs from the adult intensive care unit (ICU), from January 2012 to December 2018. The daily doses of antimicrobial administered were calculated.

**RESULTS:**

The incidence rate of UTI was 7.2/1000 patient days, with 3.5/1000 patient-days of bacteriuria, and 2.1/1000 patient-days of candiduria. Of 373 microorganisms identified, 69 (18.4%) were Gram-positive cocci, 190 (50.9%) Gram-negative bacilli, and 114 yeasts (30.7%). *Escherichia coli* and *Candida* spp. were the most common. Patients with candiduria had higher comorbidity score (Charlson Comorbidity Index ≥ 3), longer length of stay (P = 0.0066), higher mortality (P = < 0.0001) severe sepsis, septic shock, and were immunocompromised when compared with patients with bacteriuria. We observed correlation between antibiotics consumption and multidrug-resistant (MDR) microorganisms.

**CONCLUSION:**

The UTIs incidence was high and was mainly caused by Gram-negative bacteria that were resistant to common antibiotics. We observed increase in the consumption of broad-spectrum antibiotics in ICU correlating with MDR microorganisms. In general, ICU-acquired candiduria may be associated with critical illness and poor prognosis.

## INTRODUCTION

Urinary tract infections (UTI) are the most frequently reported healthcare associated infection (HAI), accounting for up to 40% of all HAIs.^1,2^ The risk of these infections increases with hospitalization in intensive care units (ICUs), where incidence rates range between 15.5% and 37.6% in low- and middle-income countries, such as Brazil.^3–5^

UTI is closely correlated with use of indwelling urinary catheters HAIs,^1,2^ and according to the Centers for Disease Control and Prevention approximately 75% of UTIs have this association.^6^ In countries, such as Brazil UTI continue to prevail and represent a major safety concern for patients.^5,7^ It is estimated that in Brazil, 16.6% to 37.6% of all ICU-acquired infections are UTI resulting in 10.7%–20.0% related deaths.^3,4,8,9^

Two important aspects about these infections are: (I) in recent years the frequent use of antibiotics in the treatment of asymptomatic infections, has resulted in the urinary tract becoming a major reservoir of resistant pathogns;^10,11^ and (II) these infections can be associated with secondary bloodstream infections (BSI), an infection that develops subsequent to a documented infection of the blood with the same organism.^12^

## OBJECTIVE

In this study, we investigated the characteristics of patients and microorganisms involved in UTIs in critically ill adult patients and the relationship of antimicrobial consumption and the number of multidrug-resistant (MDR) isolate.

## METHODS

### Patients, study, and data collection

A 7-year retrospective observational study was conducted, from January 2012 to December 2018, for the detection of patients with UTI with first episode of positive urine culture (≥ 10^5^ colony forming unite, [CFU]/mL) after 48 hours of hospitalization in adult mixed ICU (30-bed), in Brazilian tertiary-care university hospital. In this surveillance, two groups were analyzed: patients with candiduria and bacteriuria. We collected the following patient data from electronic database of clinicians and the Infection Control Service Records: age, sex, comorbidities, acute physiology score,^12^ chronic comorbidity Charlson Comorbidity Index (CCI),^13^ incidence of sepsis,^14^ and septic shock,^15^ immunosuppression (age ≥ 60, blood neoplasia, use of corticosteroids, or immunocompromising disease), use of invasive devices, length of unit stay (LOS), use of urinary catheterization, previous use of antibiotics and multidrug-resistant isolate.

### Definitions

UTI was defined as an infection in a patient using urinary catheter for a period ≥ 48 hours with positive urine culture of no more than two organisms.^16,17^ In Brazil these infections are defined in UTI and Catheter-associated Urinary Tract Infections following National Health Surveillance Agency (Agência Nacional de Vigilância Sanitária) criteria.^18^ BSI was defined by clinical criteria and documented by a positive culture result.^18,19^ The ICU-acquired positive BSI culture was considered to be associated with urinary tract infection if there was a concurrent or subsequently positive culture with the same organism within a 14-day period.^17^ MDR was defined as an acquired infection nonsusceptibile to at least one agent in three or more antimicrobial categories, including beta-lactams, aminoglycosides, and fluoroquinolones for Gram-negative bacilli (GNB); oxacillin/methicillin for *Staphylococcus* sp.; and *Enterococcus* sp.^20^ Previous use of antibiotics was considered when patients received antimicrobial therapy 72 hours after hospital admission and before the diagnosis of microbial infection.^21^ The outcomes were classified as death or survival during hospitalization; however, it was not ascertained if the death happened during their stay in the ICU.

### Clinical, microbiological, and antibiotic resistant profile

Microbial identification and antimicrobial susceptibility test were performed on a VITEK II system (bioMérieux, Brazil) for the following antimicrobials: aminoglycoside (gentamicin and amikacin), carbapenems (imipenem, meropenem, and ertapenem), cephalosporin (cefazolin, ceftriaxone, cefuroxime, and cefepime), glycopeptides (vancomycin and teicoplanin), rifampicin, fluoroquinolone (ciprofloxacin), polymyxin (E and B), and penicillin plus β-lactamase inhibitors (piperacillin-tazobactam, tetracyclines, and ampicillin-sulbactam). Data on antifungal susceptibility tests were not available. Quality-control protocols were used according to the standards of the Clinical and Laboratory Standard Institute.^22^ The isolate with intermediate susceptibility were considered resistant.^20^

### Calculation of incidence infection rates and density


$$\text{UTI}/1000\text{patient}-\text{day}=\frac{\text{Number of UTIs}}{\text{Total number of patient}-{\text{days}}^{\text{A}}}\text{x}100$$

^A^ Patient-day = P x B x O

P = Period of observation in days

B = Beds available in the unit

O = Occupancy rate in the period considered (%)

### Defined daily dose of antimicrobial (DDD) per 1000 patient-days

The most used antibiotics were selected for calculations per 1000 patient-days: cefepime, ceftriaxone, imipenem, meropenem, tigecycline, and polymyxin B. The density of DDD per 1000 patient-days was obtained by the following formula:


$$\begin{array}{l} \text{DDD}=\frac{\text{Antibiotic consumption in grams}}{{\text{Defined daily dose}}^{23}}\\ \text{DDD}/100\text{patient}-\text{days}=\frac{\text{DDD}}{\text{Total number of patient}-\text{days}}\text{x}100 \end{array}$$

### Statistical analysis

The Chi-square tests or Fisher's exact test were used to compare discrete variables. Fisher's exact test was used instead of the Chi-square test when one or more expected values in the 2 × 2 contingency table were equal or less than 5. The comparison of two quantitative variables was made using the Mann–Whitney test for nonparametric variables and the Students-*t* test for parametric variables. Two-sided tests were used for all analyses. All P value < 0.05 was considered statistically significant. The Pearson's correlation coefficient test was used to describe the relationship between antibiotic consumption and bacterial resistance rate (GraphPad Prism version 6.0 [La Jolla, California, United States]). The epidemiological data were analyzed through using the GraphPad Prism 6.0 (La Jolla, California, United States) and BioEstat 5.0 (Tefé, Amazonas, Brazil).

### Ethical considerations

Data and the samples analyzed in the present study were obtained according to the standards approved by the Institutional Ethics Committee of the Universidade Federal de Uberlândia (Protocol number 1.627.990, July 5, 2016).

## RESULTS

During a 7-year period (2012–2018), a cohort of 363 critical patients with first episode of UTI were included in the study, of these 252 (69.4%) were caused by bacteria and 109 (30.0%) by *Candida* sp. Further, two episodes of infection had a fungal etiology of the genus *Trichosporon* (0.5%), and these were not included in the comparative analyzes between bacteriurias and candidurias. The incidence rate of UTI was 7.2/1000 patient-days with 3.5/1000 patient-days for bacteriuria and 2.1/1000 patient-days for candiduria (Table 1). Overall, only 10/363 (2.7%) episodes were polymicrobial.

**Table 1:** Incidence of Urinary Tract Infections per 1.000/patient-days

Variables	Epidemiological indicators/ years							
	2012	2013	2014	2015	2016	2017	2018	Total
UTI/1.000 patient-day	3.92	5.11	4.01	8.9	14.4	7.8	6.5	**7.2**
Bacteriuria/1.000 patient-day	2.83	3.65	3.01	6.3	9.3	5.9	4.2	**3.5**
Candiduria/1.000 patient-day	1.09	1.36	0.82	2.6	5.0	1.8	2.2	**2.1**
Use of IDC (%)	95.0	93.3	80.0	89.9	93.6	92.6	95.7	**90.9**
Mortality crude (%)	23.0	37.5	31.8	44.8	39.8	20.9	37.5	**38.8**

UTI = Urinary Tract Infection; IDC = Indwelling Urinary Catheter.

The most common characteristics of the patients were: women (58.6%), clinical patient (67.7%), cardiopathy (53.1%) and nephropathy (52.0%). Majority of the patients had high acute and chronic illness severity scores with average severity index score (ASIS) ≥ 4 (74.6%) and high score for chronic comorbidity CCI (54.5%), severe sepsis (29.2%), septic shock (42.9%), and used invasive procedures and broad-spectrum antibiotics therapy (96.9%; Table 2). The average length of hospitalization and that after diagnosis were prolonged, 15 (standard deviation [SD] ± 13.29) and 11 days (SD ± 12.52), respectively. The crude mortality rate was 38.8% and was more frequently among those with candiduria (55.0%) than those with bacteriuria (31.3%; Table 2).

**Table 2:** Epidemiological characteristics of patients with first episode positive urinary culture and comparison between candiduria and bacteriuria

Characteristics		Total patients = 363	Bacteriuria n = 252	Candiduria n = 109	P^1^	OR^2^ CI-95%
**Sex**						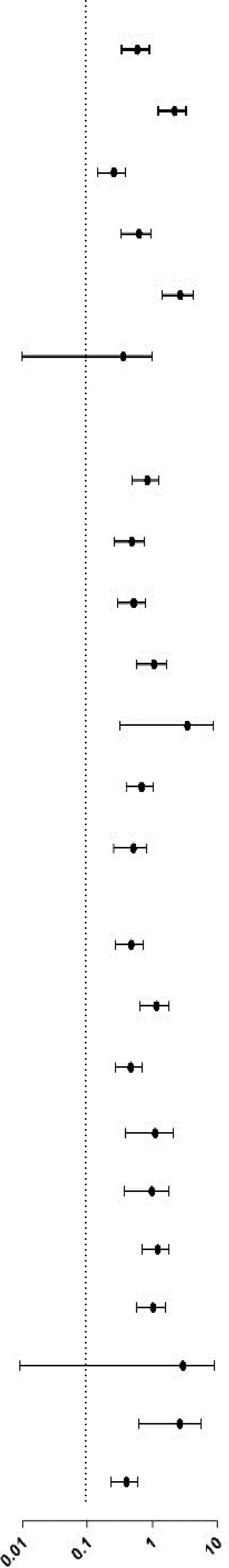
	Woman	213 (58.6)	136 (53.9)	75 (68.8)	0.0086^*^	
	Male	152 (41.8)	116 (46.0)	34 (31.1)	0.0086^*^	
**Ages, year (mean) ± SD** 3		55.91 ± 19.03	54.81 ± 18.25	58.35 ± 20.55	< 0.0001^*^	
**Patient**						
	Clinical	246 (67.7)	161 (63.8)	83 (76.1)	0.0223^*^	
	Traumatic	112 (30.8)	90 (35.7)	22 (20.1)	0.0034^*^	
	Surgical	5 (1.3)	1(0.3)	4 (3.6)	0.0146^*^	
**Comorbidities**						
	Cardiopathy	193 (53.1)	128 (50.7)	63 (57.7)	0.2209	
	Diabetes mellitus	86 (23.6)	48 (19.0)	38 (34.8)	0.0012^*^	
	Nephropathy	189 (52.0)	118 (46.8)	71 (65.1)	0.0014^*^	
	Neuropathy	89 (24.5)	61 (24.2)	28 (25.6)	0.7643	
	HIV^4^+	9 (2.4)	7 (2.7)	2 (1.8)	0.5978	
**Immunocompromised Score Clinical**		175 (48.2)	113 (44.8)	62 (56.8)	0.0356^*^	
	Asis^5^ ≥ 4	271 (74.6)	179 (71.0)	92 (84.4)	0.0070^*^	
	Charlson ≥ 3	198 (54.5)	123 (48.8)	75 (68.8)	0.0005^*^	
**Severity of infection**						
	Sepsis	106 (29.2)	74 (29.3)	32 (29.3)	0.9989	
	Septic shock	156 (42.9)	93 (36.9)	63 (57.7)	0.0002^*^	
	ICU-LOS^6^ ≥15 days	187 (51.5)	118 (46.8)	68 (62.3)	0.0066^*^	
**Invasive procedures**						
	Indwelling bladder catheter	330 (90.9)	228 (90.4)	100 (91.7)	0.7014	
	Mechanical ventilation	322 (88.7)	223 (88.4)	99 (90.8)	0.5120	
	Tracheostomy	146 (40.2)	103 (40.8)	43 (39.4)	0.8003	
	Hemodialysis	92 (25.3)	54 (21.4)	36 (33.0)	0.6714	
**IDC** 7 **(mean/days) ± SD**		13 ± 13.29	12.29 ± 0.85	14.67 ± 12.55	0.2903	
**Previous use of antimicrobials**		352 (96.9)	245 (97.2)	104 (95.4)	0.3786	
**Mortality**		141 (38.8)	79 (31.3)	60 (55.0)	< 0.0001^*^	

1 Statistically significant < 0.05;

2 OR = odds ratio; CI = confidence interval;

3 SD = standard deviation;

4 HIV = human immunodeficiency virus;

5 ASIS= Average Severity Index Score.

6 ICU-LOS = Intensive Care Unit-Length of Hospital Stay;

7 IDC = indwelling urinary catheter.

There was significant difference in patient characteristics between those with bacteriuria and candiduria. The patients with candiduria were women (68.6%, P = 0.0086), older (58.3 years, SD 58.35 ± 20.55, P = < 0.0001), had more severe illness, had diabetes mellitus (P = 0.0012) and nephropathy (P = 0.0014), with presence of septic shock (P = 0.0002). Moreover, patients with candidemia showed more ICU-LOS than those with bacteriuria, > 15 days (62.3% versus 46.8%, P = 0.0066). Furthermore, traumatic patients were most frequently observed in the group of patients with bacteriuria (P = 0.0034). In addition, high frequency of mechanical ventilation in both groups was observed; however, it was not statistically significant (Table 2). Majority of the patients were using a bladder catheter (90.9%) for a longer period, with an average of 13 days (SD ± 13.29; Table 2).

Of the 373 microorganisms identified, 69 (18.4%) were Gram-positive cocci, with a predominance of *Enterococcus faecalis* (n = 30, 43.4%) and *Staphylococcus epidermidis* (n = 12, 17.3%), 190 (50.9%) were GNB, with a predominance of *Escherichia coli* (n = 68, 35.7 %), and 114 (30.7%) were *Candida* sp. Although the number of non-fermenting GNB isolated was low (17.8), a high rate of carbapenem-resistant *Acinetobacter baumannii* (73.1%) was found as well MDR strains (63.1%). MDR rates in *K. pneumoniae* and *E. coli* were 62.2% and 32.3%, respectively. Meanwhile, the rate of multidrug resistance of Gram-positive bacteria was low (17.3%). However, 66.6% of *Staphylococcus haemolyticus* strains and 36.3% of *S. aureus* were MDR (Table 3).

**Table 3:** Pathogens responsible for positive urine culture in intensive care unit (ICU)

Microorganisms/ resistance profile		n total = 373/total MDR (%)
Gram-negative Bacilli		190 (50.9)
	carbapenem resistant/ MDR^1^	76 (40.0)/82 (43.6)
*Escherichia coli*		68/190 (35.7)
	carbapenem resistant /MDR	55 (80.8)/22 (32.3)
	ESBL^2^	5/55 (7.3)
*Klebsiella pneumoniae*		45/190 (23.6)
	*carbapenem resistant/ MDR*	4 (8.8)/28 (62.2)
	ESBL	8/45 (17.7)
*Pseudomonas aeruginosa*		15/190 (7.8)
	carbapenem resistant/ MDR	3 (20.0)/4 (26.6)
*Acinetobacter baumanni*i		19/190 (10.0)
	Carbapenem resistant/ MDR	14 (73.6)/12 (63.1)
Others^3^		43/190 (22.6)
	MDR	17/43 (39.5)
Gram-positive Cocci		69 (18.4)
	oxacillin resistant/ MDR	10 (14.4)/12 (17.3)
*Staphylococcus aureu*s		11/69 (16.0)
	oxacillin resistant/ MDR	4 (36.3)/4 (36.3)
*Staphylococcus epidermidis*		12/69 (17.3)
	*o*xacillin resistant/ MDR	2 (16.6)/2 (17.0)
*Staphylococcus haemolyticus*		6/69 (9.0)
	*o*xacillin resistant/ MDR	4 (66.6)/4 (66.6)
*Enterococcus faecalis*		30/69 (43.4)
Others^4^		11/69 (14.6)
	MDR	2/11 (20.0)
Yeasts		114 (30.7)
	*Candida albicans*	65/114 (57.0)
	*Candida tropicalis*	25/114 (21.9)
	*Candida glabrata*	9/114 (7.8)
	Others^5^	15/114 (13.1)

1 MDR = Multidrug-resistance;

2 ESBL = Extended-spectrum beta-lactamase;

3 *Klebsiella oxytoca, Enterobacter cloacae, Enterobacter gergoviae, Enterobacter aerogenes, Citrobacter koseri, Citrobacter freundii, Hafnia alvei, Serratia marcescens, Proteus mirabilis, Stenotrophomonas maltophilia, Raoultella planticola;*

4 *Sphylococccus capitis, Staphylococcus hominis, Staphylococcus saprophyticus, Enterococcus faecium, Enterococcus hirae, Streptococcus agalactiae;*

5 *Candida guillermondii, Candida krusei, Candida utilis, Candida lamata, Trichosporon sp.*

In the study 41.3% (150/363) patients had BSI, and 26.1% of these patients had the infection after the first episode of UTI. The urinary tract was the probable focus of infection, i.e., had the same microorganisms in blood and urine in only 11.5% (11/95) of these patients. These infections were caused mainly by bacteria in 7/11 (63.3%) cases, and the yeast genus *Candida* in 4/11 (26.3%) cases. It is important to highlight that among these patients who presented fungemia three died (75.0%; data not demonstrated).

**Figure 1:**
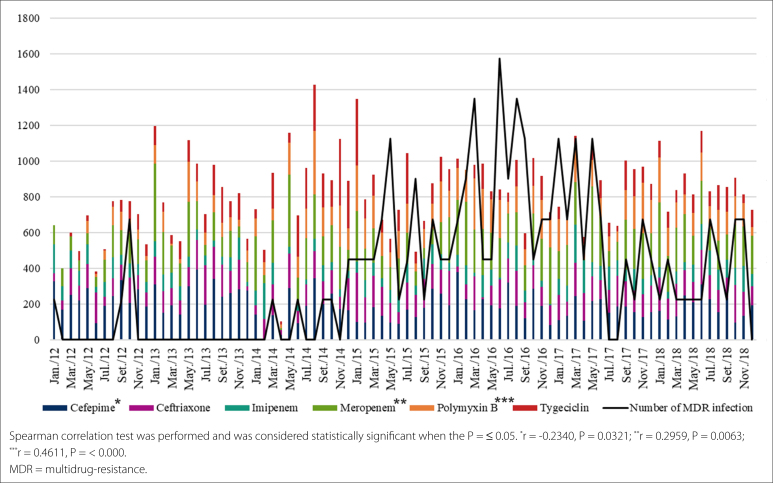
Relationship between the defined daily dose of antimicrobials per 1.000 patient-days and the number of patients with multidrug-resistant urinary tract infections per 1.000 patient day in the intensive care unit of hospital.

Figure 1 shows the relationship between the defined daily dose of antibiotics/1000 patient-days and the number of patients with MDR microorganisms UTI/1000 patient-days. A variation of 104–196.7 per 1000 patient-days in the consumption of broad spectrum cephalosporins, fluoroquinolones, ertapenem, imipenem, polymyxin, and tigecycline was observed during the study period. On the other hand, there was an increase in the consumption of meropenem (data non demonstrated). Despite this, a positive correlation was observed between the increase in MDR isolate and the consumption of meropenem (r = 0.4611 and P = 0.0063) and polymyxin (r = 0.2959 and P = < 0.0001; Figure 1).

## DISCUSSION

In this retrospective analysis, we attempted to investigate the characteristics of critical patients with candiduria and bacteriuria and the relationship of antimicrobial consumption and the number of multidrug-resistant isolate. In total, 363 cases of UTI were observed and 373 microorganisms were isolated. Our findings indicate that the presence of UTI was associated with diagnosis of admission as trauma in bacteriuria patients and older age, severe illness, diabetes mellitus, and nephropathy in candiduria patients. These infections are a problem in ICU because they are the second most important healthcare-associated infection in critically ill patients,^17^ associated with high morbidity and costs.^1–3^ The situation is more serious in countries like Brazil, which already has higher rates of HAI, in addition to the constant lack of financial resources to invest in the control and prevention of these.^3,4^

Overall, the higher UTI indicators found in this study's ICU compared with that by Aubron et al.^17^ may be due to high and inappropriate use of urinary catheter. Although there are few studies that describe the differences between bacteriuria and candiduria, in a recent study Ding et al.^1^ did not find differences between these two groups. However, our study found significant differences between them. Patients with ICU-acquired candiduria had higher comorbidities score (CCI ≥ 3), presented severe sepsis, septic shock, and were immunocompromised when compared with patients with bacteriuria. Higher incidence of mortality and a longer ICU-LOS, as reported in another study,^17^ was also observed.

The overuse and misuse of antimicrobials in hospital settings, has caused increased bacterial resistance over time, particularly in lower and middle-income countries.^3,4,17,24^ In Brazil, a rising number of scientific articles have shown high frequencies of bacterial resistance especially among infections due to *K. pneumoniae*, *P. aeruginosa,* and *A. baumannii.* ^3,4^ Historically, the literature describes the urinary tract as a reservoir of MDR microorganisms.^25,26^

These microorganisms were common in our cohort; High rate of occurrence of bacteria from the Enterobacteriaceae family and *Candida* spp., especially *C. albicans* (57.0%) was observed. This increase in fungal infections has been reported by other studies.^17^ The impact of antibiotic therapy on microbiological ecology contributes to the emergence of these pathogens. In addition, a high frequency of *E. faecalis* was also found among Gram-positive species (43.4%). This is an interesting finding, since in developing countries and particularly in Brazil, these infections are primarily caused by GNB.^24^

Moreover, in this cohort we found alarming frequencies of MDR *A. baumannii* and *P. aeruginosa* strains, as well as high intensity consumption of the broad-spectrum cephalosporins followed by carbapenems. In addition, positive correlation was found between the consumption of polymyxin B and meropenem with multidrug-resistant infections. This positive correlation between carbapenems and MDR infections was also demonstrated in other studies.^27,28^ In general, the quantity of antibiotics for general use in the evaluated ICU was higher than that compared to other countries.^29–31^ Our results reinforce that the ICU is a favorable environment for the emergence of resistant microorganisms, and it is necessary for countries to invest in strategies to prevent these infections. Likewise, the importance of urine as a source of these phenotypes has also been demonstrated.

Although unexpected, we found a high mortality rate in our cohort (38.8%); however, this was attributed to several factors, such as BSI occurring concomitantly or after UTIs and the severity of acute clinical diseases. As previously mentioned, *Candida* sp. were very common in our investigation and patients with candiduria had higher mortality rates. Thus, our results suggest an association between candiduria and increased patient morbidity, which is likely to be a marker of patient severity, as noted by Horan et al.^16^ In general, our results indicate that efforts to prevent nosocomial UTI (candiduria or bacteriuria) are required as although they are often not considered serious infections, our findings show otherwise. Likewise, it is extremely necessary and urgent to implement protocols for the conscious consumption of antimicrobials which are evaluated frequently as high consumption rates of these drugs and alarming rates of MDR pathogens have been observed. This reinforces the need for governments to invest in surveillance and control of these infections in developing countries.

## CONCLUSION

In conclusion, the data presented in this report fortify the fact that UTIs caused by MDR GNB organisms and *Candida* sp. in adult ICUs are a challenge for the patient safety. The UTI rates and the consumption of antimicrobials found in our study were higher than that from of countries. Better strategies for the effective and systematic surveillance and prevention of this problem is required for greater adherence to infection control measures and antimicrobials use in ill patients.
